# Glycosylation-Dependent Induction of Programmed Cell Death in Murine Adenocarcinoma Cells

**DOI:** 10.3389/fimmu.2022.797759

**Published:** 2022-02-10

**Authors:** Aleksei Parshenkov, Thierry Hennet

**Affiliations:** Institute of Physiology, University of Zurich, Zurich, Switzerland

**Keywords:** lectin, knockout, apoptosis, necroptosis, pyroptosis, autophagy

## Abstract

Altered surface glycosylation is a major hallmark of tumor cells associated with aggressive phenotype and poor prognosis. By recognizing specific carbohydrate motifs, lectins can be applied to distinguish tumor from healthy cells based on the expression of glycosylation-dependent markers. Through their ability to bind to specific carbohydrates, lectins induce cell agglutination and cross-link surface glycoproteins, thereby mediating mitogenic and death-inducing effects in various cell types. The carbohydrate-selective cytotoxic effect of lectins also enables their possible application in therapies targeting cancer cells. To clarify the intracellular pathways mediating cell death induced by a group of plant and fungal lectins, we investigated mouse adenocarcinoma MC-38 cells harboring inactive genes involved in apoptosis, necroptosis and pyroptosis. Treatment of MC-38 cells with wheat germ agglutinin, *Maackia amurensis* lectin I, and *Aleuria aurantia* lectin induced multiple cell death pathways through reactions that relied on the autophagy machinery without depending on caspase activation. Furthermore, inhibition of *de novo* protein synthesis by cycloheximide strongly decreased the cytotoxic response, indicating that the lectins investigated induced cell death *via* effector molecules that are not expressed under normal circumstances and supporting the non-apoptotic nature of cell death. The broad cytotoxic response to lectins can be beneficial for the development of combination therapies targeting tumor cells. Given that tumors acquire resistance to various cytotoxic treatments because of mutations in cell death pathways, compounds inducing broad cytotoxic responses, such as lectins, represent potent sensitizers to promote tumor cell killing.

## Introduction

Glycosylation is a complex post-translational modification involved in the regulation of multiple cellular reactions, such as proliferation, adhesion, and trafficking among others. Evidence shows that glycosylation also plays a role in regulating cell death through multiple pathways, including but not limited to prevention of death receptor internalization as in the case of the Fas and TNFR1 receptors, or enhancement of death receptor sensitivity to cognate ligands followed by activation of cell death programs (1–6). Altered glycosylation, such as characterized by increased fucosylation and sialylation of surface glycoproteins, is a hallmark of cancer and is considered as a target for development of diagnostic and therapeutic tools (7–9). The vast structural diversity of glycosylation is paralleled by a similarly diverse group of carbohydrate-binding proteins, referred to as lectins, which occur in all types of organisms, from bacteria and fungi up to plants and animals. The recognition of specific carbohydrate motifs by lectins enabled their application to distinguish tumor from healthy cells based on the expression of glycosylation-dependent tumor markers (10–14). Several plant and fungal lectins represent promising molecules to target and eliminate various tumors (10, 12, 13, 15).

In addition to their applications as cell markers, lectins exert mitogenic and death-inducing effects on various cell types. For example, concanavalin A and phytohemagglutinin activate T-lymphocytes by crosslinking glycosylated signaling receptors (16). The same concanavalin A induce apoptosis in cancer cells through selective crosslinking and inhibition of receptor tyrosine kinases (17, 18). Because of differences in surface glycosylation between tumor and normal cells, lectins can be applied alone or in a combination with other therapeutic agents to induce cell death selectively in cancer cells while keeping healthy cells intact (12, 19–21). Cell death induced by lectins follow different pathways, such as apoptosis, paraptosis-like death, autophagy and programmed necrosis (10, 19, 20, 22, 23). Specific lectins induce distinct modes of cell death in different type of tumor cells. Wheat germ agglutinin, for example, induces apoptosis in melanoma and leukemic cell (20, 21), whereas it kills cervical carcinoma cells through paraptosis-like cell death (19).

While targeting surface glycans is a promising approach for cancer therapies, lectin-based approaches are still at an early stage of development, as shown in clinical trials with mistletoe lectin (10, 24). The development of efficient therapies based on glycan targeting requires a deep understanding of the mechanisms of cell death induced by lectins on target tumor cells. Using a murine adenocarcinoma cell model, the present study addresses the multiple cell death pathways activated in response to treatment of cells with lectins targeting different glycan structures. Despite targeting different glycan motifs, the lectins tested induced similar pathways of cell death, which were caspase-independent and relied on *de novo* protein synthesis.

## Materials and Methods

### Cell Culture

Mouse colon adenocarcinoma MC-38 cells were cultured in Dulbecco’s modified Eagle’s medium (DMEM) supplemented with 0.1 mM non-essential amino acids and 10% FBS. Human embryonic kidney HEK293T cells were cultured in DMEM supplemented with 10% FBS. Young adult mouse colon (YAMC) cells were cultured in DMEM supplemented with 2% FBS, 0.2 μM progesterone, 0.224 μM sodium selenite, 10 μg/ml insulin, 100 μg/ml transferrin, 0.49 μM triiodothyronine, 0.45 μM L-thyroxine, and 5 units/ml mouse interferon-gamma, and incubated at the proliferation permissive temperature of 33°C (25).

### CRISPR/Cas9-Mediated Gene Knockout in MC-38 Cells

Single-guide RNAs (sgRNAs) targeting genes of interest in MC-38 cell line were designed using CRISPOR online tool (26). sgRNAs were cloned into either lentiCRISPRv2-puro (Addgene plasmid #52961) or lentiCRISPRv2-neo (Addgene plasmid #98292) (27, 28), lentivirus was produced in HEK293T cells using polyethylenimine “MAX” transfection reagent (Polysciences) followed by transduction of MC-38 cells with lentiviral vectors according to protocols from the Zhang laboratory (27, 29). LentiCRISPRv2-puro transduced MC-38 cells were selected using 10 µg/ml puromycin in DMEM, lentiCRISPRv2-neo transduced cells were selected with 1.25 mg/ml G-418 (Thermo Fischer Scientific) for a time when non-transduced control contained no viable cells. Single clones of transduced cells were obtained by limiting dilutions. Knockouts were validated at the genomic level by PCR and at the protein level by Western blotting (**Supplementary Figure 1**). GNE knockout was validated by genomic PCR followed by Sanger sequencing and by staining with mannose-binding Concanavalin A (ConA) as a control of glycosylation unaffected by GNE knockout, MAL II, and ECL followed by flow cytometry analysis (**Supplementary Figure 2**). The sequences of sgRNAs and corresponding primers for validation of knockouts by PCR are listed in **Table 1**. To avoid clonal effects, two validated clones were used for each knockout. More than one sgRNA sequence per gene listed in **Table 1** indicates that clones were generated using different sgRNAs to minimize potential off-target effects.

**Table 1:** sgRNA sequences for targeted gene inactivation in MC-38 using CRISPR/Cas9 system and corresponding primers for knockout verification by PCR.

Gene	Exon	sgRNA sequence	Forward primer	Reverse primer
*BAX*	3	AGCGAGTGTCTCCGGCGAAT	CTTGGTTCTCAACATTCTGCTCCT	GGATTCTATCTGAGTTGAGTGGAGG
*BAK1*	3	GGGGCAAGTTGTCCATCTCG	TCATGTGCCAGGACTAACTCTCA	GTAGGGATGAGCATCAGTCAGAGA
	4	GGAACTCTGTGTCGTAGCGC	GAGCCCTATCAGACCTTCAGACA	GAAGTTGGTATGTTCACCCTGACAC
*TRADD*	2	AGCCGGTCAGAATGGCCACG	ACTTTTTGTTAAAGGCAATGGAGGG	CACAAAGTCCCAGAGTCACTACAC
	3	CCTCCAAGCCTACCGCGAGG	GACTATGGGCTTAGCTTTCTCCTC	ATGTAATTCAAACAGCGCTCTTCAT
*FADD*	1	TAGATCGTGTCGGCGCAGCG	CGATCTGATGGAGCTCAAGTTCT	GTAAGAAACAAGACCTCCCAGCTT
	2	CCGGACTGGTTAAGGCGCTG	GGCATTTGACATTGTGTGTGACAA	TACATCATGGTGTGATCAAGTCCAC
*CASP8*	3	CTTCCTAGACTGCAACCGAG	TTTATGCTATTGCTGAAGAACTGGG	TGTATTTAGCCCCTACATTTAGCCC
*RIPK3*	3	GTGGGACTTCGTGTCCGGGC	CTTCCAGAGCGCAATCCAATTTT	CAGAATGTTAGAGGGCTTGAGGTC
*MLKL*	2	GCACACGGTTTCCTAGACGC	GATACACAGGGGATTGTGGTATTTC	CATGGAAGAGGATCTTATCATTGCC
	2	GACTTCATCAAAACGGCCCA	GATACACAGGGGATTGTGGTATTTC	CATGGAAGAGGATCTTATCATTGCC
*CASP1*	5	GAGGGCAAGACGTGTACGAG	AACAAGGTTGGTTTCTTGAAAGGAC	AGAAGTTTTACCAGAGCTGTGAGAT
*GSDMD*	4	GCAACAGCTTCGGAGTCGTG	ACTTCTCCGTGTTTGAACTTGTCAT	CTTAGTAGAGTCTTCCACCACTGC
*GNE*	5	AGGAGATGGTTCGAGTGATG	TATCAGCTCTTGGATGAGATGCAG	GTAGGTACCGGTTTCTCTTCCTATC
	9	GATCCAGGAATGGAACTCCG	TTCACTCAGAACTGTCTGATTCCTT	CAGTTCTGGTACACCCTGAAGAAC

### Lectin Treatment

MC-38 cells were dissociated with 2 mM EDTA in PBS (pH 7.4) and resuspended in DMEM supplemented with 5% FBS. In total, 1.5 x 10^4^ cells in 100 µl of the medium were seeded per well of 96-well plate followed by incubation for 3 h at 37°C to allow cells to adhere to the well surface. Afterward, 50 µl of cell medium in every well were replaced with 50 µl of DMEM containing 2x concentrations of either lectin, in the presence or absence of cycloheximide (Santa Cruz Biotechnology), or corresponding controls followed by incubation at 37°C for 20 h. Final concentrations of MAL I, AAL, WGA were 50 µg/ml, 60 µg/ml, and 4 µg/ml, respectively. Cycloheximide was used at 2.5 µg/ml final concentration. An equivalent amount of lectin resuspension buffer was used as a negative control, recombinant mouse TNF-α (BioLegend) at 20 ng/ml and 80 µM cisplatin (Santa Cruz Biotechnology) were used as positive controls for apoptotic cell death. Cell viability was assessed in a fluorescence-based assay using double staining with Hoechst 33342 (Thermo Fischer Scientific) and propidium iodide (Stemcell Technologies). In experiments, in which cycloheximide was used, cytotoxicity assay based on lactate dehydrogenase (LDH) release was performed instead. All lectins were from Vector Laboratories (Burlingame, California, USA).

### Cell Death Measurement

Cytotoxicity was measured either by fluorometric method based on double staining with propidium iodide (PI) and Hoechst 33343 (Hoechst) or by LDH cytotoxicity WST assay (Enzo Life Sciences) according to the manufacturer’s protocol. For the fluorometric assay, 11 µl of DMEM containing 10x concentrations of Hoechst (10 µg/ml for MC-38 and 30 µg/ml for YAMC cells) and PI (50 µg/ml) were added to each well of 96-well plate 30 min before the end of treatment. Cells were washed once with 100 µl Dulbecco’s phosphate-buffered saline (DPBS). Another 100 µl of DPBS were added per each well followed by fluorescence measurement at 535/617 nm for PI and 360/460 nm for Hoechst using a plate reader (Tecan Infinite® 200 Pro). Before calculating cytotoxicity PI/Hoechst ratio was determined. Cytotoxicity was calculated using the following equation:


$$Cytotoxicity\;(\%)=\frac{PI/Hoechst(test\;sub\tan ce)-PI/Hoechst(low\;control)}{PI/Hoechst(high\;control)-PI/Hoechst(low\;control)}\times 100$$

Positive control values were measured as a mean PI/Hoechst value from three independent experiments by treating MC-38 WT or YAMC cells with 20 ng/ml mouse TNF-α (BioLegend) in combination with 2.5 µg/ml cycloheximide (Santa Cruz Biotechnology). The PI/Hoechst ratio from untreated cells was used as a negative control. For LDH assays, supernatants from lysed cells treated with cycloheximide were used as positive controls.

### Flow Cytometry

Totally, 2 x 10^5^ cells were stained with 200 µl of 10 µg/ml fluorescein isothiocyanate or biotin-conjugated MAL I, MAL II, SNA, AAL, WGA, and ECL (Vector Laboratories) in lectin staining buffer (PBS, 1% FBS, 0.1 mM CaCl_2_) for 30 min on ice followed by washing twice with the same buffer. When using biotinylated lectins, additional incubation with streptavidin-FITC (Biolegend) for 20 min on ice was performed. Before the analysis using a FACScanto II flow cytometer (BD Bioscience), cells were washed twice and resuspended in lectin staining buffer.

### Western Blotting

Cells were lysed using RIPA buffer (50 mM Tris-HCl pH 8.0, 150 mM NaCl, 0.1% Triton X-100, 0.5% sodium deoxycholate, 0.1% SDS) and total protein concentration was quantified using Pierce™ BCA Protein Assay Kit (Thermo Fisher Scientific). In total, 20 µg of total protein of each sample were separated using 12% SDS-polyacrylamide gel electrophoresis followed by wet transfer to 0.2 µm nitrocellulose membrane for LC3A/B or 0.45 µm membranes for other targets with subsequent incubation with primary and secondary antibodies and detection using ECL reagent (Thermo Fischer Scientific). The following antibodies were used: anti-MLKL (37705S), anti-TRADD (3694S), anti-BAK (3814S), anti-BAX (2772S), anti-caspase-8 (4927S), anti-cleaved caspase-8 (9429S), anti-caspase-3 (9662S), anti-PARP (9542S), anti-caspase-9 (9504S), anti-LC3A/B (4108S) were from Cell Signaling Technology (Danvers, Massachusetts, USA); anti-FADD (ab124812), anti-GSDMD (ab209845), anti-GAPDH (ab9485), goat anti-rabbit IgG (HRP) (ab205718) were from Abcam; anti-RIPK3 (NBP1-77299) was from Novus Biologicals (Littleton, Colorado, USA); anti-caspase 1 (14–9832–82), goat anti-rat IgG2a (HRP) (PA1-84709) were from Thermo Fisher Scientific (Waltham, Massachusetts, USA). For quantitative Western blotting of LC3A/B, protein levels were normalized to total proteins as measured by staining nitrocellulose membranes with Ponceau S (0.2% Ponceau S, 3% trichloroacetic acid, 3% sulfosalicylic acid) for 3 min shortly after the transfer followed by wash with deionized water. All the band intensities were quantified using ImageJ software.

### Caspase Activity and Inhibition Assay

Activity of caspases- 3/7 was measured by cleavage of the fluorescently labeled substrate Ac-DEVD-AFC (Sigma-Aldrich). The assay was performed as previously described (30). Briefly, 30 μl of cell lysates was mixed with 30 μl of assay buffer containing 100 µM of respective substrate in black opaque 96-well plate followed by 30 min for caspase-3/7 substrate or 60 min incubation for caspase-8 and -9 substrates at 37°C followed by fluorescence reading at 400/505 nm using a plate reader (Tecan Infinite® 200 Pro). For inhibition assays, 1.5 x 10^4^ cells in DMEM, 5% FBS were seeded in wells of 96-well plates 3 h before lectin treatment. The broad-spectrum pan-caspase inhibitor Q-VD-OPh (Sigma-Aldrich) was added at 20 μM 1 h before lectin treatment and its concentration was maintained at the same level after addition of lectins.

### Statistical Analysis

One-way ANOVA followed by Bonferroni’s test was used to assess significance between multiple experimental conditions. Unpaired two-tailed t-test was applied to determine significance between two experimental conditions. Differences were considered statistically significant for *p < 0.05*. The error bars in the figures represent the standard deviation (SD). Prism 9.2.0 Software (GraphPad) was used for statistical analysis.

## Results

### Glycan Ligands and Cell Death Induced by Lectins

The key property of lectins is the ability to recognize specific glycan structures in terms of their monosaccharide composition and glycosidic linkage. In this study, we used a panel of six lectins of plant and fungi origin featuring distinct glycan specificities and containing at least two carbohydrate-recognition domains enabling the crosslinking of cell surface targets. This panel consisted of *Aleuria aurantia* lectin (AAL), which recognizes fucose-containing glycans (31), *Maackia amurensis* lectin I (MAL I), binding to glycans containing β1-4 galactose and α2-3 sialic acid (32), *Maackia amurensis* lectin II (MAL II), which recognizes α2-3-linked sialic acid (32), *Sambuccus nigra* agglutinin (SNA), which recognizes α2-6-linked sialic acid (33), wheat germ agglutinin (WGA), which binds to glycans containing N-acetylglucosamine and sialic acid (34), and *Erythrina crystagalli* lectin (ECL), which preferentially binds to terminal β1-4 galactosylated residues (35). Considering that the abundance of sialic acid-terminated glycans on cancer cells is often associated with a poor prognosis and thus considered to be a prospective target for anticancer therapies, we investigated the impact of MC-38 desialylation on the binding ability and cytotoxic effect of lectins recognizing sialylated and non-sialylated glycan epitopes. For that purpose, we established MC-38 cell line lacking expression of UDP-N-acetylglucosamine 2-epimerase/N-acetylmannosamine kinase (GNE), the rate-limiting enzyme of sialic acid biosynthesis.

**Figure 1:**
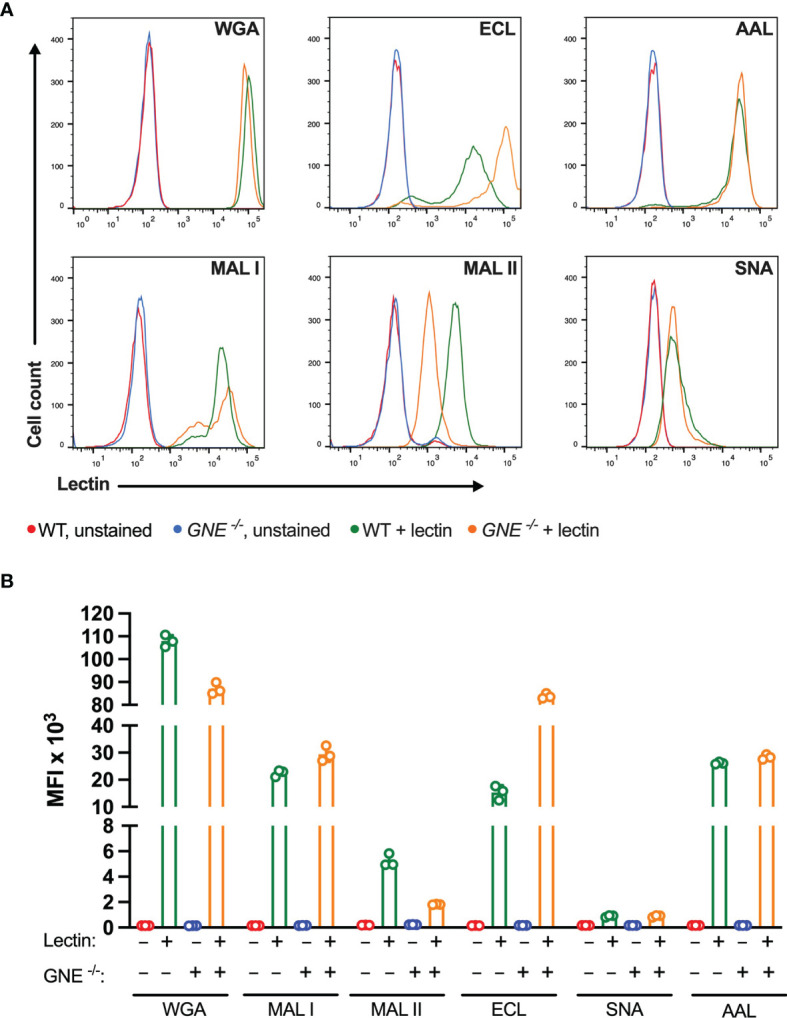
Binding of plant lectins to MC-38 cells. **(A, B)** Binding ability of lectins used in the study to MC-38 cells with unaffected membrane sialylation level (MC-38 WT) and desialylated (MC-38 *GNE -/-*) measured by flow cytometry. Unstained MC-38 WT (red lines), stained MC-38 WT (green lines), unstained MC-38 *GNE -/-* (blue lines), stained MC-38 *GNE -/-* (orange lines). Each lectin was used at 10 µg/ml. Data are presented as mean and standard deviation of three replicates.

Among the lectins tested, WGA showed the highest binding levels to MC-38 cells, indicating a wide availability of N-acetylglucosamine ligands exposed at the cell surface. WGA is also known to bind to sialylated glycans (36), yet MC-38 lacking GNE only showed minimally decreased binding for WGA (**Figure 1**). The weaker binding of ECL, AAL, MAL I and MAL II reflected lower densities of cognate carbohydrate ligands. As expected, GNE^-/-^ MC-38 cells showed decreased binding of α2-3-sialic acid-specific MAL II. By contrast, the low binding of SNA was not affected by GNE inactivation, thereby showing that either α2-6-sialylated ligands are largely absent on MC-38 cells or SNA lectin still able to recognize a minimal levels of sialic acids maintained by the lysosomal salvage pathway of sialic acids (37). Furthermore, while SNA is considered as a very specific lectin, non-specific binding is still possible to occur (38). Interestingly, ECL binding was stronger in GNE^-/-^ cells, which expose terminal β1-4 galactose in the absence of sialic acid capping.

**Figure 2:**
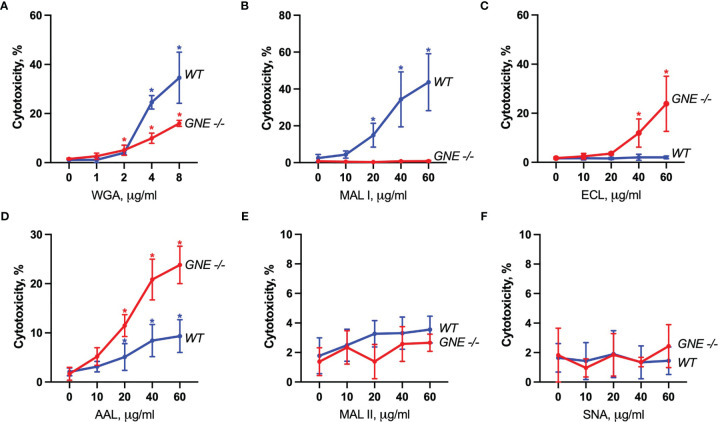
Lectin-induced cell death in MC-38 cells. Cytotoxicity was measured by propidium iodide and Hoechst 33342 staining of cells after treatment with WGA **(A)**, MAL I **(B)**, ECL **(C)**, AAL **(D)**, MAL II **(E)**, and SNA **(F)** lectins in WT and GNE deficient cells for 20 h. Data represent averages and SD of 6 replicates out of 2 independent experiments. *p < 0.05; one-way ANOVA followed by Bonferroni’s multiple comparison test.

The cytotoxic response of the lectins tested largely matched the density of their carbohydrate ligands. MAL I, AAL, WGA in wildtype (WT), and ECL, AAL in GNE^-/-^ cells, induced cell death in a dose-dependent manner with WGA showing the highest cytotoxic effect (**Figure 2**). Despite the minimal decrease in WGA binding observed in GNE^-/-^ cells, the cytotoxic effect of WGA was significantly lower in desialylated cells (**Figure 2A**). This observation is in line with reports on human leukemia cells, which were less sensitive to WGA-mediated cytotoxicity when treated with neuraminidase (20). The cytotoxic effect of MAL I was completely abolished in GNE^-/-^ cells (**Figure 2B**), although MAL I binding was similar between WT and GNE^-/-^ cells (**Figure 1**). This finding underlines the importance of glycan composition for the cytotoxic effect mediated by MAL I, which likely relies on the cross-linking of sialylated glycoproteins by MAL I. The opposite effect was observed for ECL, which was only cytotoxic towards GNE^-/-^ cells (**Figure 2C**). The increase in cytotoxicity matched the increased lectin binding measured in GNE^-/-^ cells (**Figure 1**), suggesting that the higher density of exposed β1-4 galactose was responsible for the sensitivity to ECL-mediated cytotoxicity. AAL showed the lowest toxic effect in MC-38 cells (**Figure 2D**) despite the strong binding of this lectin to cells (**Figure 1**), thus showing that the cross-linking of fucosylated glycoproteins does not significantly induce cell death. Surprisingly, the lack of sialic acids in GNE^-/-^ cells resulted in much stronger cytotoxicity mediated by AAL (**Figure 2D**). The sialic-acid dependent lectins MAL II and SNA, which recognize other sets of sialylated glycans than MAL I, also failed to induce a cytotoxic response in MC-38 cells over the range of concentrations tested (**Figures 2E, F**). Considering the lower cytotoxicity mediated by WGA and MAL I in GNE^-/-^ cells (**Figures 2A, B**), sialylation, however, appears to be critical for the cytotoxic effect of the latter lectins. Accordingly, the lack of cytotoxicity associated with MAL II and SNA indicates that binding to sialylated ligands alone is not sufficient to induce cell death. The activation of cell death pathways probably requires cross-links between differentially glycosylated surface proteins.

### Lectins Activate Multiple CellDeath Pathways

**Figure 3:**
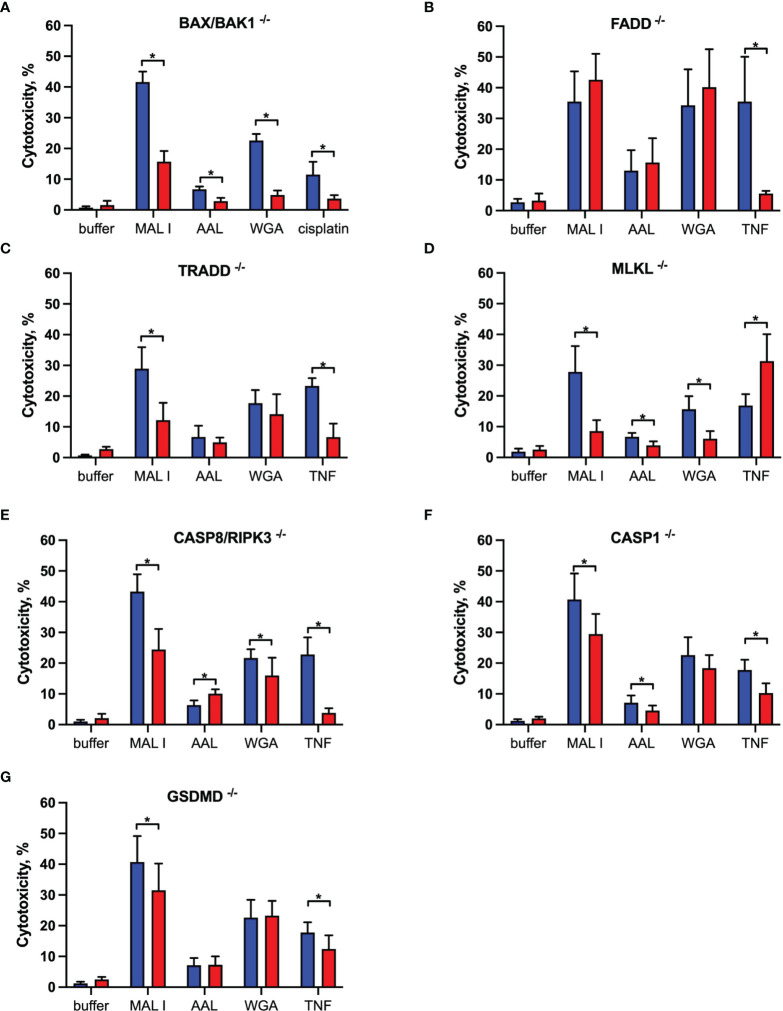
Effects of single gene knockouts on lectin-induced cell death in MC-38 cells. Intrinsic apoptosis was assessed by inactivation of BAK/BAX **(A)**, whereas extrinsic apoptosis and necroptosis pathways were assessed by inactivation of FADD **(B)**, TRADD **(C)**, MLKL **(D)**, and combined caspase 8/RIPK3 **(E)** genes. Pyroptosis was assessed by caspase 1 **(F)** and GSDMD **(G)** gene inactivation. To exclude clonal effects, all experiments were performed in at least two clones for each gene knockout. Data represent 6 replicates from at least 2 independent experiments. WT (blue bars), gene knockouts (red bars). *p < 0.05; unpaired two-tailed t-test.

To characterize the signaling pathways mediating cell death induced by the cytotoxic lectins WGA, MAL I and AAL, we used a panel of MC-38 cells with knockouts in genes involved in cell death responses. Apoptosis is the most common form of programmed cell death induced in response to various extrinsic and intrinsic stimuli. The extrinsic apoptosis pathway is induced in response to activation of cell death receptors, followed by formation of the death-inducing signaling complex (DISC) that includes Fas-associated *via* death domain protein (FADD) and pro-caspase-8, which cleaves the executioner caspase-3. The intrinsic apoptosis signaling cascade is activated in response to various internal cell stress factors, such as DNA damage, and results in the formation of pores in outer mitochondrial membranes. The pro-apoptotic proteins BCL2 antagonist/killer 1 (BAK1) and BCL2 associated X (BAX) form these pores, which lead to the release of cytochrome C and activation of initiator caspase-9, which in turn activates the executioner caspase-3 (39, 40). We first addressed the role of BAX and BAK1, the members of pro-apoptotic BCL-2 protein family mediating intrinsic apoptosis (40). The inactivation of the BAX/BAK1 complex decreased the cytotoxic response induced by WGA, MAL I and AAL treatment (**Figure 3A**). The decrease in cytotoxicity by more than 50% was similar to the effect achieved in cells treated with cisplatin, which is a classical trigger of apoptosis. By contrast, the inactivation of FADD (41), an adaptor protein involved in the extrinsic signaling pathway of apoptosis, did not impacted the cell death mediated by WGA, MAL I and AAL, whereas tumor necrosis factor-α (TNF)-induced cell death was impaired as expected (**Figure 3B**).

Another mode of programmed cell death is necroptosis, which is caspase-independent and is mediated by tumor necrosis factor receptor type 1-associated death domain protein (TRADD), receptor-interacting serine/threonine protein kinases 1 (RIPK1) and 3 (RIPK3) and mixed lineage kinase domain-like protein (MLKL) in response to extrinsic stimuli. MLKL, when phosphorylated by RIPK3, affects cell membrane permeability resulting in membrane rupture and necroptotic morphology (42). The inactivation of TRADD (43), another adaptor molecule required for activation of apoptosis and necroptosis downstream of tumor necrosis factor receptor 1 (TNFR1), decreased cell death in cells treated with MAL I, but not when WGA and AAL was added (**Figure 3C**). This observation suggests that MAL I could potentially mediate its cytotoxic effects *via* crosslinking of TNFR1 or other receptors, such as death receptor 3, that are also known to induce cell death *via* TRADD (44). To assess the ability of the lectins to induce necroptosis, we inactivated MLKL, which is essential in the execution of necroptosis. The loss of MLKL decreased the cytotoxic effect of WGA, MAL I and AAL, indicating the contribution of the necroptosis pathway in cell death induced by these lectins (**Figure 3D**). Of note, loss of MLKL resulted in increased sensitivity to TNF that shows a potential role of MLKL in preventing non-necroptotic forms of cell death induction in response to TNF in MC-38 cells. Given that apoptosis and necroptosis could be activated by similar stimuli, as in case of TNF/TNFR1 signaling, and considering inhibitory effect of caspase-8 on activation of necroptosis that plays a role in switching of apoptotic cell death to necroptosis, we tested the combined role of caspase-8 and RIPK3 in cell death mediated by lectins (40, 42, 45, 46). MC-38 with caspase-8/RIPK3 double knockout showed substantial decrease in cell death in MAL I-treated cells and minor decrease in WGA-treated cells that serves as an indicator of activation of either extrinsic apoptosis or necroptosis in treated cells (**Figure 3E**). Concurrent involvement of MLKL in MAL I and WGA-mediated cell death also confirms a potential activation of necroptosis in response to lectin treatment (**Figures 3D, E**).

The third mode of programmed cell death investigated in this study is pyroptosis. The main pathway of pyroptosis is mediated by caspase-1, which is activated by the inflammasome in response to various microbial infections and non-infectious stimuli. Pyroptosis is central in immune cells, such as macrophages (47), but can also occur in other cell types in response to infection or exposure to chemical compounds (48, 49). The pathway leads to gasdermin D (GSDMD) cleavage, which embeds in the plasma membrane and forms pores that disrupt ionic gradients and facilitate water influx, hence leading to cell swelling and osmotic lysis (47, 50). Considering that some plant lectins are known to induce formation of the NLRP3 inflammasome in immune and cancer cells (51), we addressed the role of pyroptosis in mediating cell death in MC-38 cells treated with WGA, MAL I and AAL. Inactivation of either caspase-1 or GSDMD in MC-38 showed only a minor decrease in MAL I-mediated cytotoxicity, supporting a partial involvement of pyroptosis in response to MAL I treatment (**Figures 3F, G**). Inactivation of caspase-1 resulted in decreased cytotoxicity induced by MAL and also by AAL (**Figure 3F**). A similar effect was observed in TNF-treated cells, in which caspase-1 and GSDMD knockouts reduced cell death, thus pointing to the induction of the pyroptosis response downstream of TNFR1.

**Figure 4:**
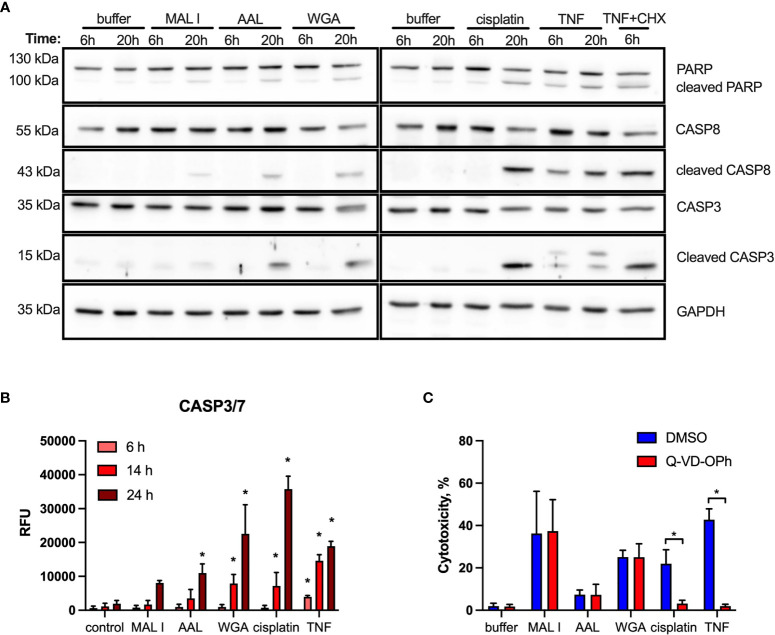
Cleavage of PARP, caspase 8 and caspase 3 induced by lectin treatment of MC-38 cells. **(A)** Immunoblot analysis of poly-ADP-ribose polymerase 1 (PARP), procaspase 8, cleaved caspase 8, caspase 3 and GAPDH as a loading control in MC-38 lysates after treatment with either lectins or cisplatin and TNF controls for 6 and 20 h** (B)** Activation assay for caspases-3,7 (DEVDase activity) using fluorogenic substrate. **(C)** Impact of caspase inhibition on cell death. MC-38 cells were treated either with DMSO or Q-VD-OPH broad spectrum caspase inhibitor in combination with lectins and respective controls. Data represent 6 replicates out of 2 independent experiments. *p < 0.05; one-way ANOVA with Bonferroni’s multiple comparison test **(B)** or unpaired two-tailed t-test **(C)**.

Given the dominant apoptosis response induced by lectins, we addressed the activation of key apoptotic caspases and the cleavage of poly (ADP-ribose) polymerase-1 (PARP1), which is a substrate of activated caspase-3 and -7 (52). Indeed, a moderate cleavage of caspases-3, -8 and PARP1 was observed in MC-38 cells after AAL and WGA treatment, while MAL I treatment only resulted in minor cleavage of the same substrates (**Figure 4A**). In comparison to lectin treatment, addition of TNF to MC-38 cells led to significant cleavage of caspases-3, 8 and PARP1 already by 6 h of treatment, while cisplatin treatment showed substantial detection of cleaved forms by 20 h. Caspase-9 cleavage was not detected, neither in cells treated with lectins, nor in cells treated with TNF and cisplatin. To confirm the validity of the anti- caspase-9 antibody applied, we tested lysates of mouse embryonic fibroblasts treated with cytochrome C, which resulted in the detection of caspase-9 cleavage products (**Supplementary Figure 3**). The comparison with TNF demonstrated that AAL and WGA treatments activated apoptotic signaling, yet in a moderate and delayed manner.

In addition to immunoblotting, we measured activation of effector caspases-3/7 using a specific fluorogenic substate. In case of MAL I treatment, no caspase activation was detected, whereas AAL treatment resulted in increased caspases-3/7 activity by 24 h of treatment. WGA treatment also yielded increased caspases-3/7 activation, which was already significant by 14 h (**Figure 4B**). To further validate the role of caspases in lectin-mediated cell death, we applied the pan-caspase inhibitor Q-VD-OPh in cells treated with WGA, MAL I and AAL. Surprisingly, Q-VD-OPh treatment did not affect the cytotoxic response of the three lectins, whereas it completely prevented cell death induced by cisplatin and TNF as expected (**Figure 4C**). This finding showed that, while activated in response to lectins, caspase activities are not essential in mediating the cytotoxic response to WGA, MAL I and AAL.

### Lectin Treatment Up-Regulates Autophagy

**Figure 5:**
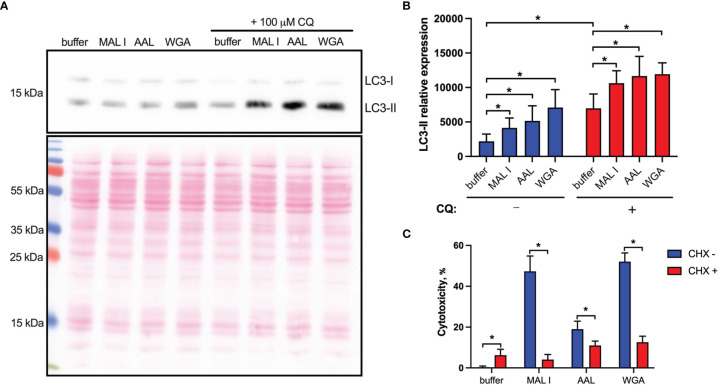
Non-apoptotic effects of lectins in MC-38 cells. **(A)** Induction of autophagy in cells treated with lectins was measured by quantitative immunoblot analysis. MC-38 cells were treated with lectins with or without chloroquine (CQ) (100 μM) for 6 h followed by lysis with RIPA buffer and analysis by immunoblot staining. Chloroquine was added 4 h prior to cell lysis **(B)** Quantification of immunoblot data by densitometry image analysis using ImageJ software. LC3-II levels were quantified and normalized to total protein levels using Ponceau S staining. **(C)** Assessment of *de novo* protein synthesis requirement for cell death induction. MC-38 cells were treated with lectins either alone or in combination with cycloheximide (2.5 µg/ml) for 20 h followed by cytotoxicity measurement using LDH release assay. All data are from at least 6 replicates out of 2 independent experiments with four **(B)** or three **(C)** technical replicates each. *P < 0.05; unpaired two-tailed t-test.

In addition to the induction of apoptosis, many lectins also up-regulate autophagy, which in some cases results in autophagy-dependent cell death. To address the possible activation of autophagy in MC-38 cells treated with WGA, MAL I and AAL, we measured the cleavage of the autophagy marker LC3. The LC3 protein is cleaved into LC3-I immediately after synthesis and is later conjugated with phosphatidylethanolamine, thereby forming LC3-II, which is associated with autophagosome membranes (53). Given that autophagy is a dynamic process, cellular LC3-II levels reflect the balance between synthesis and degradation resulting from the fusion of autophagosomes with lysosomes. To minimize LC3-II degradation, chloroquine was added to inhibit autolysosome formation and lysosomal protease activity (54, 55). Treatment with MAL I, AAL and WGA up-regulated autophagy in MC-38 cells, as shown by immunoblotting of LC3-II in lysates from cells treated for 6 h with lectins in the absence and presence of chloroquine (**Figures 5A, B**). The increase in steady-state LC3-II levels detected in the absence of chloroquine treatment together with concurrent involvement of BAK1 and BAX, which are known to affect lysosomal and autolysosomal permeability, indicated that cell death induced by MAL I, AAL and WGA was probably initiated through activation of the autophagic/lysosomal response rather than the classical apoptotic mitochondrial pathway (56).

### Lectin-Mediated Cell Death Depends on *De Novo* Protein Synthesis

Another cell death pathway reported to be activated in tumor cells in response to WGA is paraptosis, which is caspase-independent and relies on *de novo* protein synthesis (19, 47). Paraptosis can be activated by oxidative stress and other mechanisms resulting in osmotic dysregulation (47, 57). Considering that autophagy also requires *de novo* protein synthesis (39), we tested whether protein synthesis is essential for execution of cell death induced by lectins by treating MC-38 cells with cycloheximide. In addition to inhibiting protein synthesis, cycloheximide is also known to block starvation-induced autophagy *via* activation of mTORC1 signaling (58). The addition of cycloheximide indeed strongly decreased the cytotoxic response of the three lectins investigated (**Figure 5C**). This finding further supported the caspase-independent nature of lectin-mediated cell death induction in MC-38 cells.

### Glycan Binding and Cell Death Induced by Lectins in Non-Transformed YAMC Cells

**Figure 6:**
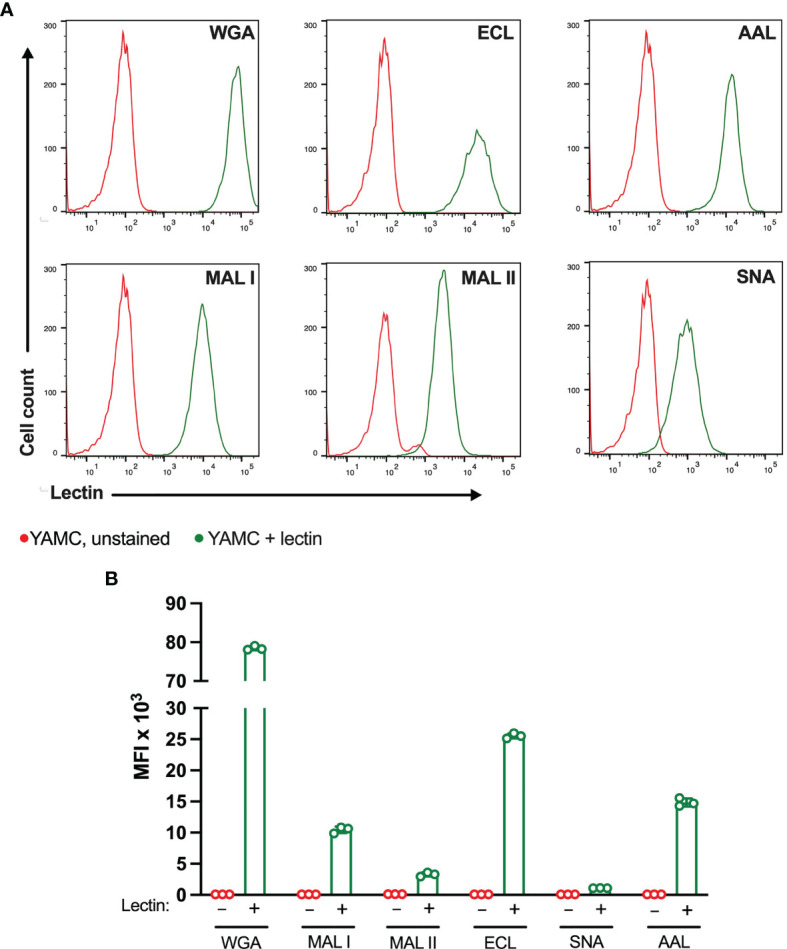
Binding of plant lectins to non-transformed YAMC cells. **(A, B)** Binding ability of lectins used in the study to YAMC cells measured by flow cytometry. Unstained YAMC (red lines), stained YAMC (green lines). Each lectin was used at 10 μg/ml. Data are presented as mean and standard deviation of three replicates.

The ability of lectins to bind and induce cell death was also evaluated in matched non-transformed colon YAMC cell line (**Figure 6**). As in MC-38 cell line, all lectins showed binding to YAMC cell surface glycans. The strongest binding was observed for WGA followed by ECL. MAL I, MAL II, AAL and SNA showed weaker binding that reflects various densities of cognate glycan structures. Compared to lectin binding to MC-38 cells, binding of WGA, ECL and SNA was noticeably higher in YAMC cells (**Supplementary Figure 4**).

**Figure 7:**
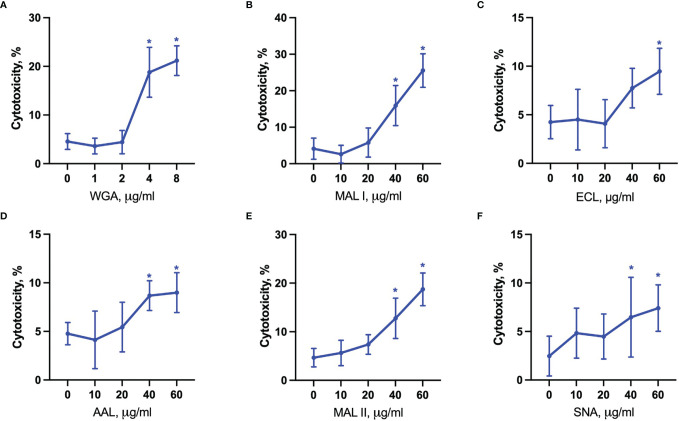
Lectin-induced cell death in YAMC cells. Cytotoxicity was measured by propidium iodide and Hoechst 33342 staining of cells after treatment with WGA **(A)**, MAL I **(B)**, ECL **(C)**, AAL **(D)**, MAL II **(E)**, SNA **(F)** lectins in YAMC for 20 h at 33°C. Data represent averages and SD of 6 replicates out of 2 independent experiments. *p < 0.05; one-way ANOVA followed by Bonferroni’s multiple comparison test.

The evaluation of cytotoxic responses mediated by lectins showed that all lectins used in the study mediated cytotoxic effects in a dose-dependent manner in YAMC cells. While ECL, AAL and SNA showed similar minimal levels of cell death, MAL I, MAL II and WGA showed the highest toxic effects (**Figures 7A–F**). It is unlikely that MAL II-mediated cytotoxicity observed only in YAMC cells was caused by various availability of glycans containing α2-3-linked sialic acids compared to MC-38 cells. The relative abundance of α2-3-linked sialic acid containing glycans was similar in YAMC and MC-38 cells as measured by flow cytometry and, therefore, variability in MAL II-meditated responses could be explained by possible resistance of MC-38 cells to programmed cell death induced by MAL II (**Supplementary Figure 4**). Altogether these results demonstrate that binding ability of all lectins and toxic effects of MAL I, AAL, and WGA are consistent in both MC-38 and YAMC cells.

## Discussion

Glycosylation is an important player in the regulation of cells death. Here, we demonstrated that MAL I, AAL and WGA did induce programmed cell death in MC-38 cell line in a caspase-independent manner, with involvement of multiple death signaling pathways, including components of apoptosis, necroptosis, pyroptosis, and autophagy. Furthermore, for MAL I- and WGA-mediated cytotoxicity, we observed the importance of sialic acid in mediation of programmed cell death by these lectins. Sialic acid may increase the binding of the corresponding lectins surface glycoproteins, thereby potentiating their cytotoxic effect. The absence of cytotoxic effects mediated by lectins that recognize sialic acid-containing glycan structures, namely MAL II and SNA, in MC-38 indicates that only glycans with certain linkage of sialic acids and the type of underlying sugars could be associated with lectin-mediated cytotoxicity in MC-38 cells. The observation of increased AAL toxicity in desialylated cells may indicate that negatively charged sialic acids may alter cytotoxicity of lectins which recognize other sugars by potentially affecting their ability to cross-link cell surface glycans while not having any negative impact on their binding ability. Moreover, the fact that ECL was only cytotoxic towards desialylated cells underlined the relevance of sialic acid removal, such as achieved by neuraminidase treatment, when targeting tumor cells (59).

One of the main features of cancer cells is their ability to resist to activation of programmed cell death *via* multiple mechanisms, including altered glycosylation and upregulation of pro-survival signaling pathways (60, 61). Compared with MC-38 cells, elevated cytotoxic responses of SNA and ECL correlated with their stronger binding to YAMC cells that indicates that lectin-mediated cytotoxicity could be dependent on the relative lectin binding strength. Among lectins used, WGA showed the highest binding ability in both YAMC and MC-38 cells with the lowest concentrations required for cell death induction that further supports the correlation between the ligand availability and cytotoxic response. Cytotoxicity of MAL II in YAMC was similar to WGA and MAL I cytotoxic responses but it did not correlate with its similar binding abilities in both cell lines which could potentially indicate an acquired resistance of cancerous MC-38 cells toward programmed cell death mode activated by this lectin.

**Figure 8:**
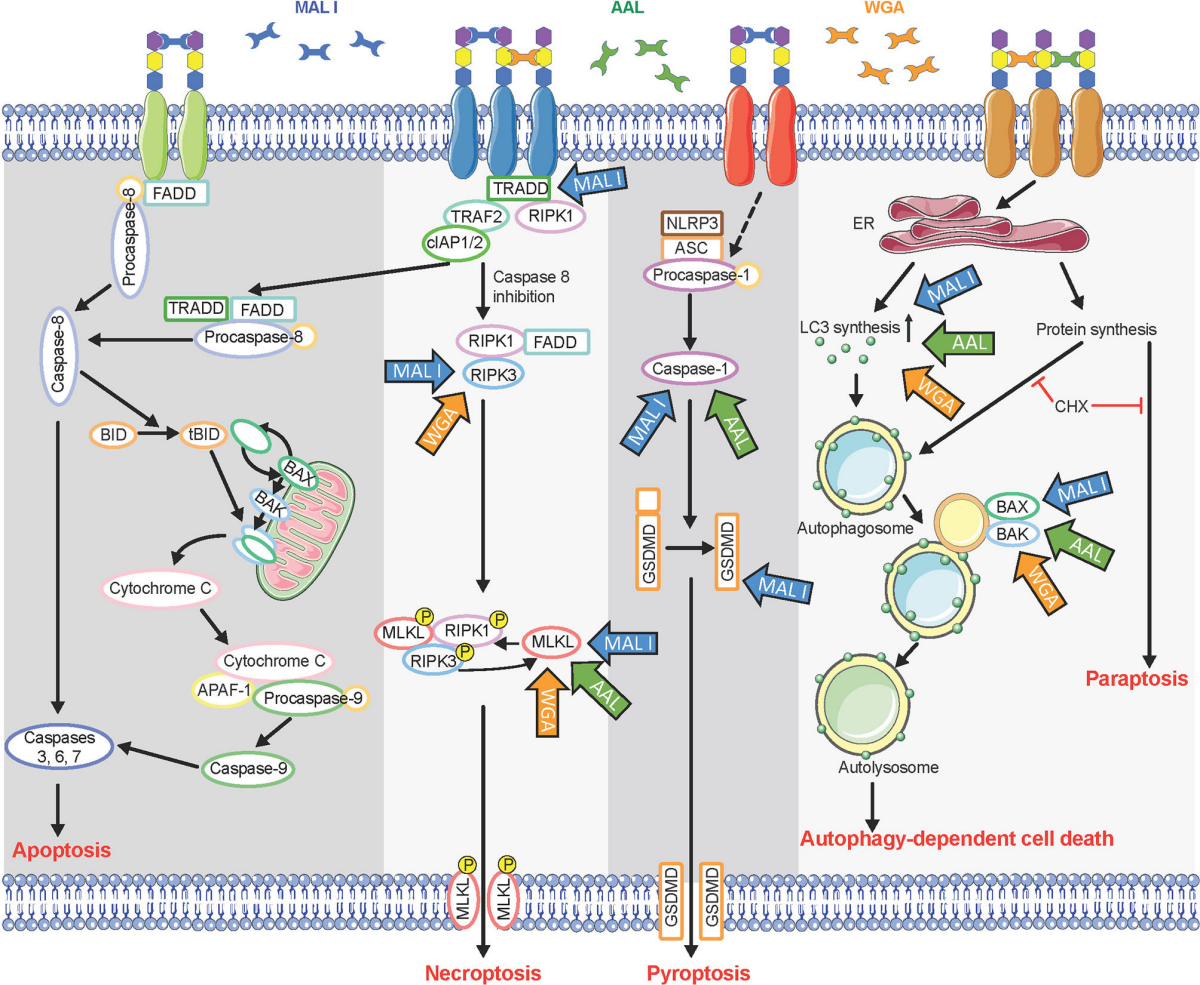
Multiple cell death signaling pathways activated in response to lectin treatment. Glycan chains on surface glycoproteins are represented by colored hexagons. Blue, green, and orange arrows indicate components of cell death signaling pathways involved in the cytotoxic effects of MAL I, AAL, and WGA, respectively. CHX, cycloheximide.

Whereas we found that MAL I, AAL and WGA induced caspase-independent cell death, we also observed that programmed cell death induced in response to lectins relied on the pro-apoptotic BCL2 proteins BAK1 and BAX. These pro-apoptotic BCL2 proteins are known for their involvement in intrinsic apoptosis through formation of pores in the outer mitochondrial membrane (39, 62). In addition to their pore-inducing effect, BAK1 and BAX have also been shown to contribute to the execution of autophagic cell death by affecting permeability of lysosomes and autolysomes (56). Increased LC3-II levels in lectin-treated cells confirmed the increased synthesis of autophagy-related membranes and at the same time reduced degradation of LC3-II located on the inner side of autophagosomal membranes. Reduced LC3-II degradation could result from autophagy inhibition through alteration of lysosomal pH, as achieved by BAK1 and BAX ability to affect permeabilization of autolysosomes. The absence of caspase 9 cleavage as an indicator of the activation of intrinsic mitochondrial cell death, which is BAK1/BAX-dependent, further supports the notion that cell death in response to MAL I, AAL and WGA lectins relies on autophagic/lysosomal alterations rather than mitochondrial damage (**Figure 8**).

The involvement of MLKL, a main executioner of necroptosis, in cell death induced by the three lectins suggests a partial contribution of the necroptosis pathway in the process. After activation by RIPK3, MLKL oligomerizes and triggers permeabilization of the cell membrane (42). In addition to its role in necroptosis, MLKL also influences non-necroptotic pathways (63). But considering the inhibitory effect of cycloheximide on the cell death, necroptosis is unlikely a key pathway mediating cell death in lectin-treated cells.

In addition to sharing common features, such as caspase-independence and need for *de novo* protein synthesis, the cell death pathways induced by MAL I, AAL and WGA presented some differences as well. For example, loss of TRADD and caspase-8/RIPK3 only affected the cell death induced by MAL I, which pointed to a prevalence of the extrinsic pathway contributing to the cytotoxic effect of that lectin. Also, the contribution of GSDMD in the cytotoxic response was also unique to MAL I. Previous studies of Maackia amurensis agglutinin, which consists of both MAL I and MAL II, did not discriminate effects of these two lectins (22, 32, 64, 65). In the study, the results obtained showed that only MAL I, and not MAL II, induces cell death in MC-38 cells, while in YAMC cells both lectins showed similar levels of toxicity. The reasons for the lack of cytotoxicity of MAL II in MC-38 remains unknown but may be related to insufficient cross-linking of glycoproteins triggering a cell death response or to the inhibition of key signaling molecules required for the activation of MAL II-mediated cell death.

The lectins applied in this study induced programmed cell death in MC-38 cell line through multiple cell death pathways, which was expected considering the structural diversity of cell surface glycoconjugates able to initiate a cell death response when crosslinked. Whereas several receptor glycoproteins require clustering for activation, membrane glycosphingolipids, known to be essential components of lipid rafts, may also contribute to the cytotoxic effects of lectins as previously reported (66, 67). Considering that tumors often acquire resistance to various cytotoxic treatments because of mutations in cell death pathways or over-expression of survival signaling pathways, compounds inducing broad cytotoxic responses can be beneficial for the development of combination therapies. Therefore, lectins could be used as potent sensitizers for killing tumors that acquired resistance to apoptosis and other cell death pathways.

## Data Availability Statement

The original contributions presented in the study are included in the article/**Supplementary Material**. Further inquiries can be directed to the corresponding author.

## Author Contributions

TH designed the study and secured the funding. AP planned and performed the experiments. AP and TH wrote the manuscript.

## Funding

This work was supported by the Swiss National Foundation grant 314730_172880.

## Conflict of Interest

The authors declare that the research was conducted in the absence of any commercial or financial relationships that could be construed as a potential conflict of interest.

## Publisher’s Note

All claims expressed in this article are solely those of the authors and do not necessarily represent those of their affiliated organizations, or those of the publisher, the editors and the reviewers. Any product that may be evaluated in this article, or claim that may be made by its manufacturer, is not guaranteed or endorsed by the publisher.

## Supplementary Material

Supplementary Figure 1 Validation of gene knockouts in MC-38 by Western blot analysis. Immunoblot analysis of MC-38 lysates harboring knockouts in **(A)** BAX and BAK1, **(B)** FADD, **(C)** TRADD, **(D)** MLKL, **(E)** RIPK3 in mouse embryonic fibroblasts (MEF) and caspase-8 deficient MC-38 cells, **(F)** Caspase-8, **(G)** Caspase-1, and **(H)** GSDMD genes. Red text indicates clones that have been used in the study. Images were acquired using either LAS-4000 (Fujifilm Life Science, Cambridge, USA) (panels **A, B, D–G)** or Fusion FX7 EDGE (Vilber, Marne-la-Vallée, France) (panels **C, H**) Western blot imaging systems.

Supplementary Figure 2 Validation of GNE gene knockout in MC-38 by flow cytometry using ConA, MAL II, and ECL. GNE knockout was performed using sgRNAs targeting either exon 5 (clone 1) **(A)**, or exon 9 (clone 2) **(B)**. Each lectin was used at 10 μg/ml.

Supplementary Figure 3 Verification of caspase 9 cleavage in MC-38 treated with lectins. **(A)** Immunoblot of caspase 9 in MC-38 treated with lectins and the positive controls cisplatin and TNF. **(B)** Validation of caspase 9 cleavage in MC-38 treated with cisplatin, equivalent volume of deionized water and mouse embryonic fibroblasts (MEF) untreated and treated with cytochrome C.

Supplementary Figure 4 Binding of plant lectins to non-transformed YAMC cells using same flow cytometer settings as for MC-38. **(A)** Binding ability of lectins used in the study to YAMC cells measured by flow cytometry. Unstained YAMC (red lines), stained YAMC (green lines). **(B)** Comparison of lectin mean fluorescence intensities in MC-38 and YAMC. Each lectin was used at 10 μg/ml. Data are presented as mean and standard deviation of three replicates.
