# Supplementary figures and images for: Glycosylation-Dependent Induction of Programmed Cell Death in Murine Adenocarcinoma Cells
Source: Front Immunol. 2022 Feb 10;13:797759. doi: 10.3389/fimmu.2022.797759 (PMC8866831; doi:10.3389/fimmu.2022.797759)

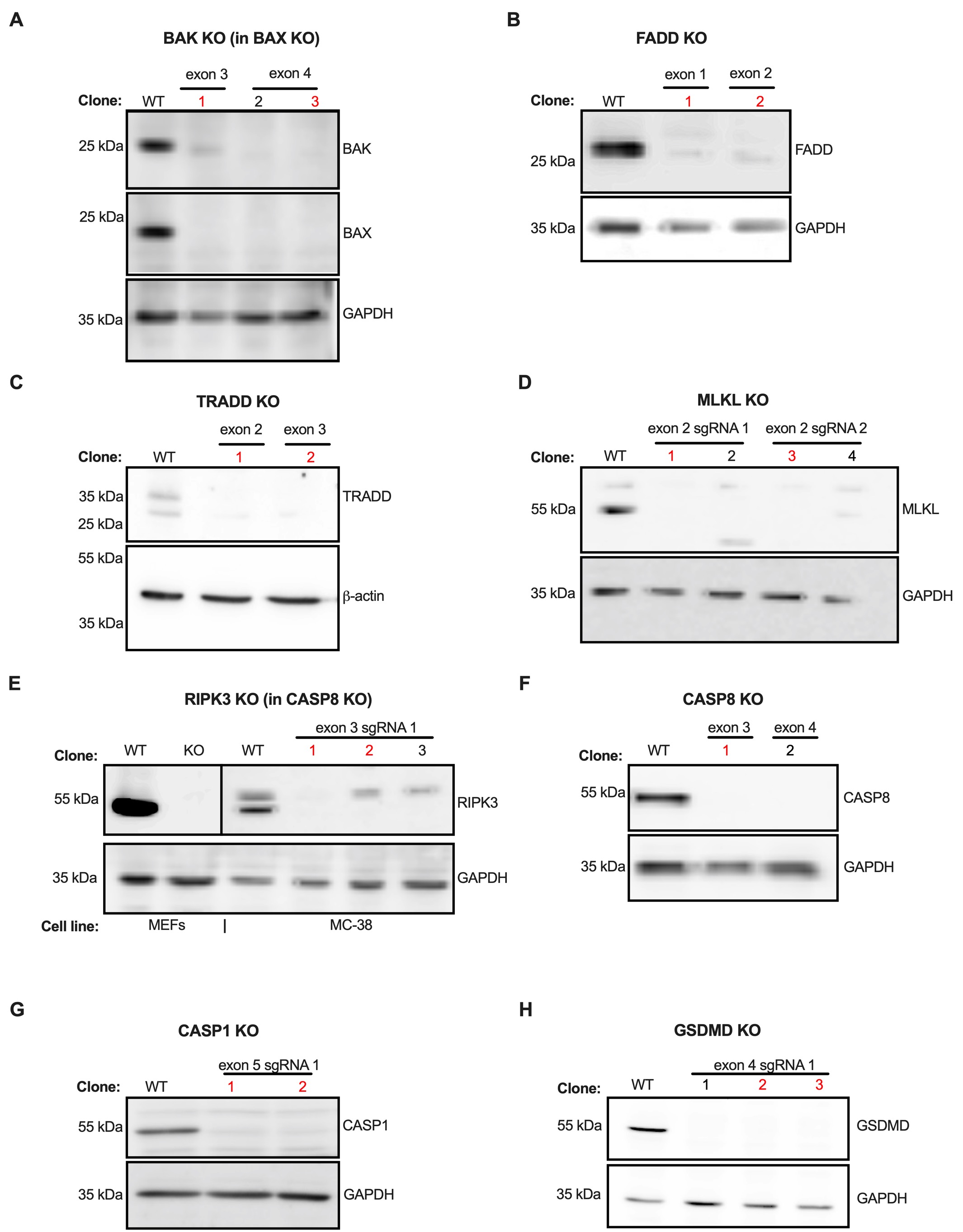

Supplement: Supplementary Figure 1 — Validation of gene knockouts in MC-38 by Western blot analysis. Immunoblot analysis of MC-38 lysates harboring knockouts in (A) BAX and BAK1, (B) FADD, (C) TRADD, (D) MLKL, (E) RIPK3 in mouse embryonic fibroblasts (MEF) and caspase-8 deficient MC-38 cells, (F) Caspase-8, (G) Caspase-1, and (H) GSDMD genes. Red text indicates clones that have been used in the study. Images were acquired using either LAS-4000 (Fujifilm Life Science, Cambridge, USA) (panels A, B, D–G) or Fusion FX7 EDGE (Vilber, Marne-la-Vallée, France) (panels C, H) Western blot imaging systems. [file Image_1.jpeg]

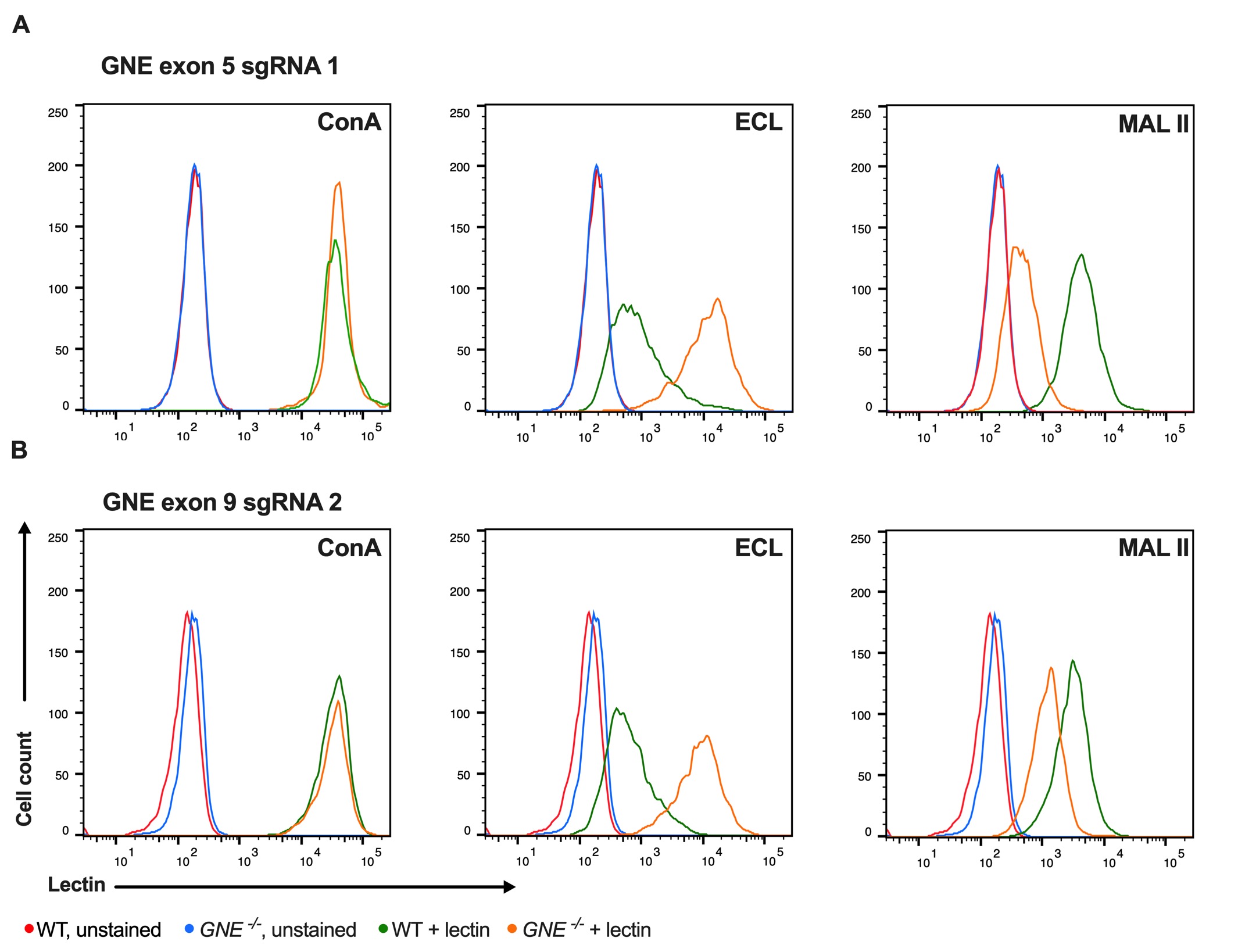

Supplement: Supplementary Figure 2 — Validation of GNE gene knockout in MC-38 by flow cytometry using ConA, MAL II, and ECL. GNE knockout was performed using sgRNAs targeting either exon 5 (clone 1) (A), or exon 9 (clone 2) (B). Each lectin was used at 10 μg/ml. [file Image_2.jpg]

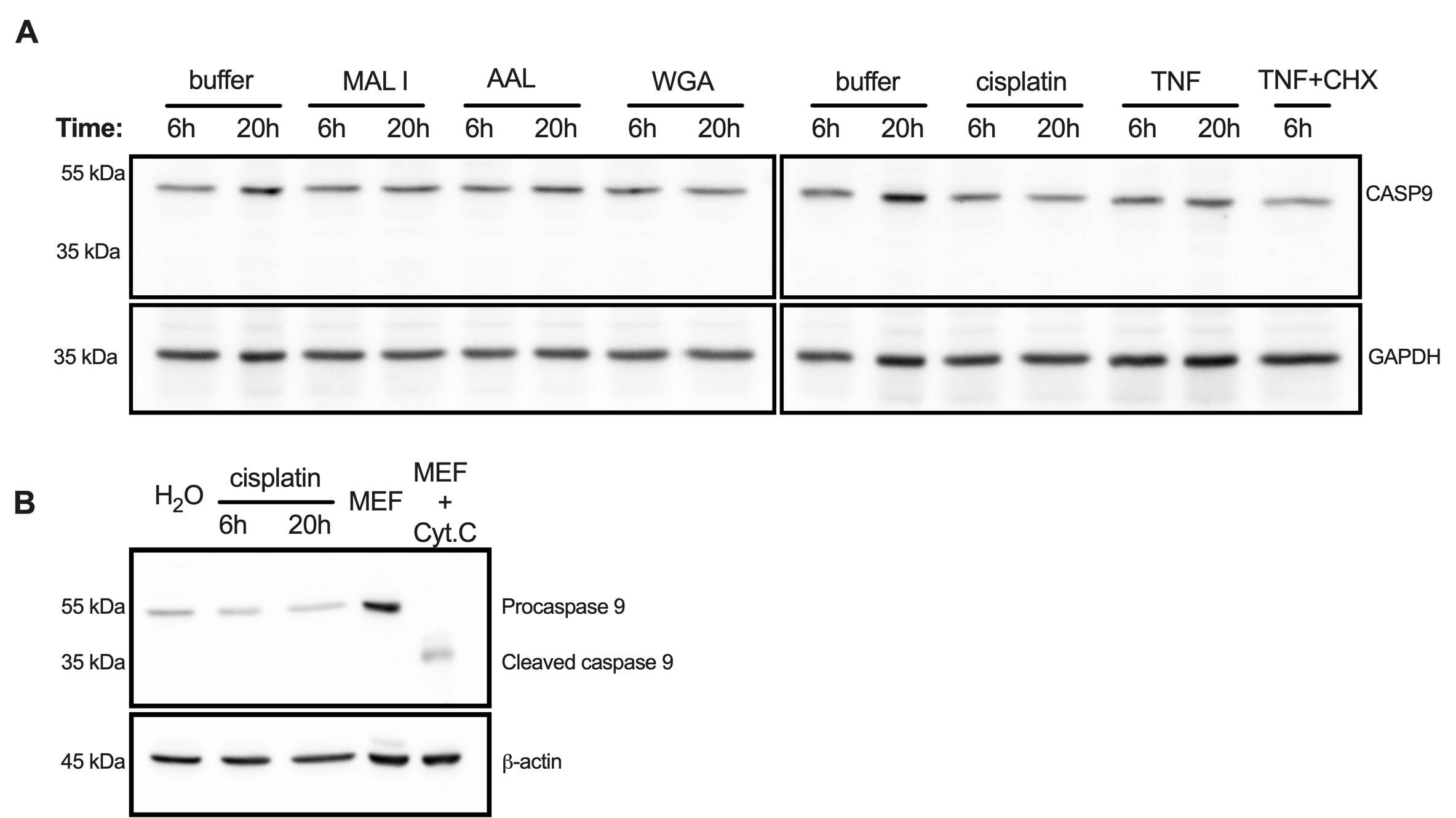

Supplement: Supplementary Figure 3 — Verification of caspase 9 cleavage in MC-38 treated with lectins. (A) Immunoblot of caspase 9 in MC-38 treated with lectins and the positive controls cisplatin and TNF. (B) Validation of caspase 9 cleavage in MC-38 treated with cisplatin, equivalent volume of deionized water and mouse embryonic fibroblasts (MEF) untreated and treated with cytochrome C. [file Image_3.jpeg]

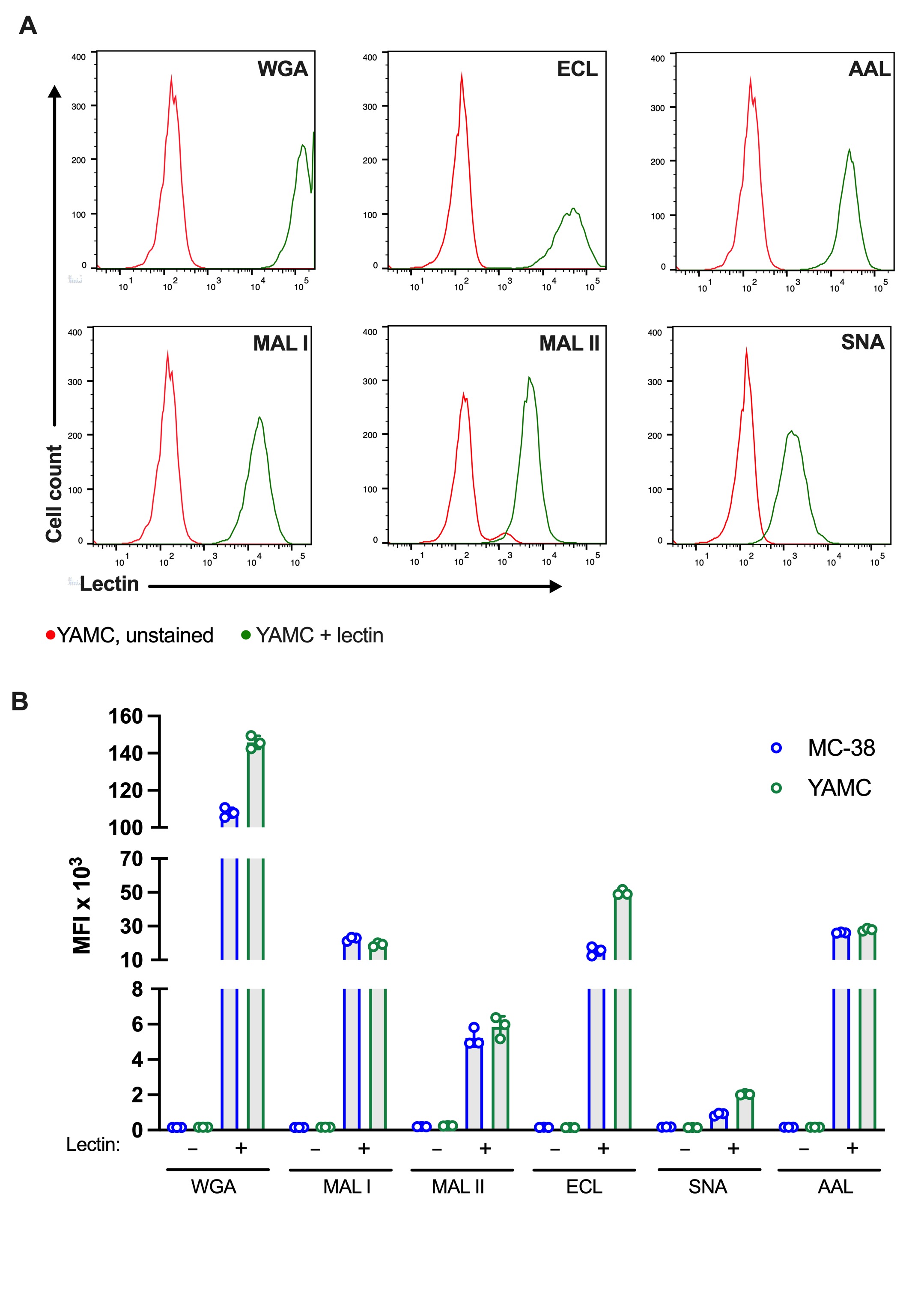

Supplement: Supplementary Figure 4 — Binding of plant lectins to non-transformed YAMC cells using same flow cytometer settings as for MC-38. (A) Binding ability of lectins used in the study to YAMC cells measured by flow cytometry. Unstained YAMC (red lines), stained YAMC (green lines). (B) Comparison of lectin mean fluorescence intensities in MC-38 and YAMC. Each lectin was used at 10 μg/ml. Data are presented as mean and standard deviation of three replicates. [file Image_4.jpeg]
